# Supplementary material for: A role for ColV plasmids in the evolution of pathogenic Escherichia coli ST58
Source: Nat Commun. 2022 Feb 3;13:683. doi: 10.1038/s41467-022-28342-4 (PMC8813906; doi:10.1038/s41467-022-28342-4)
Supplement: Supplementary file 1 — Supplementary Information [file 41467_2022_28342_MOESM1_ESM.pdf]

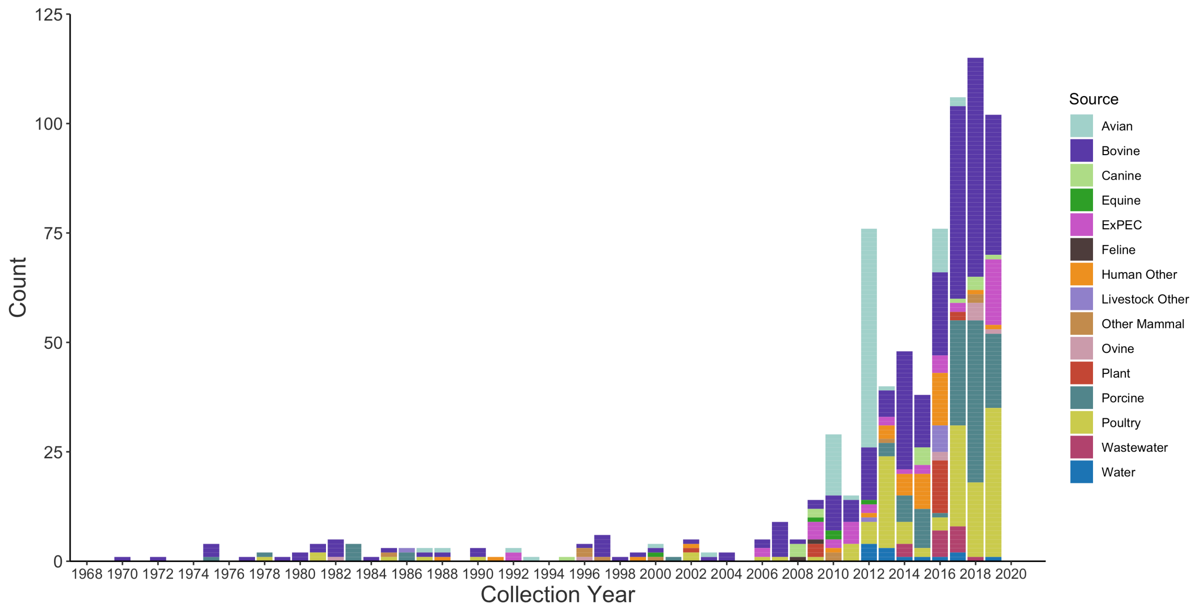

Figure S1. Temporal distribution of ST58 by source. Full distribution of sample collection years stratified by source for 752 ST58 strains.

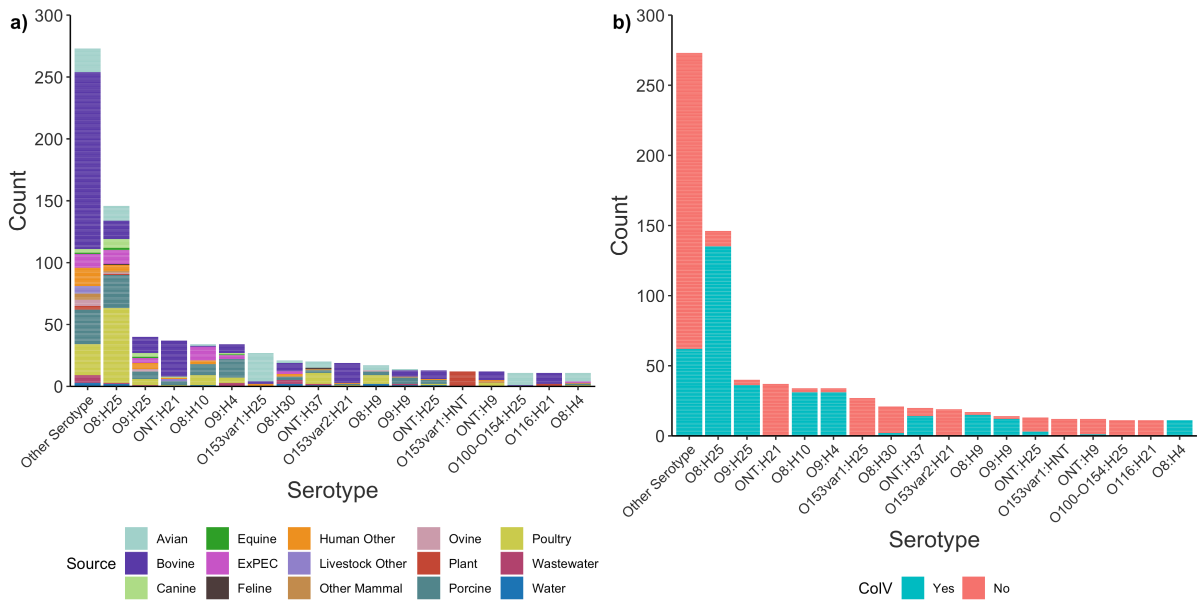

Figure S2. Relationship between serotypes, sources and ColV carriage. Distribution of serotypes stratified by **a)** source and **b)** ColV carriage.

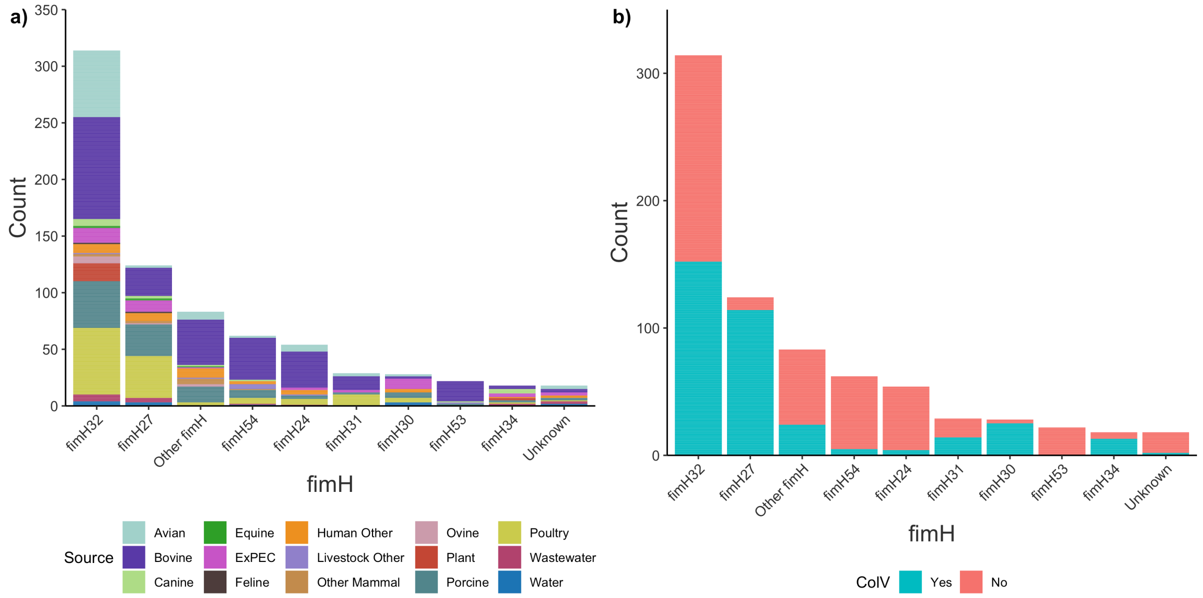

Figure S3. Relationship between *fimH*, sources and ColV carriage. Distribution of fimH alleles stratified by **a)** source and **b)** ColV carriage.

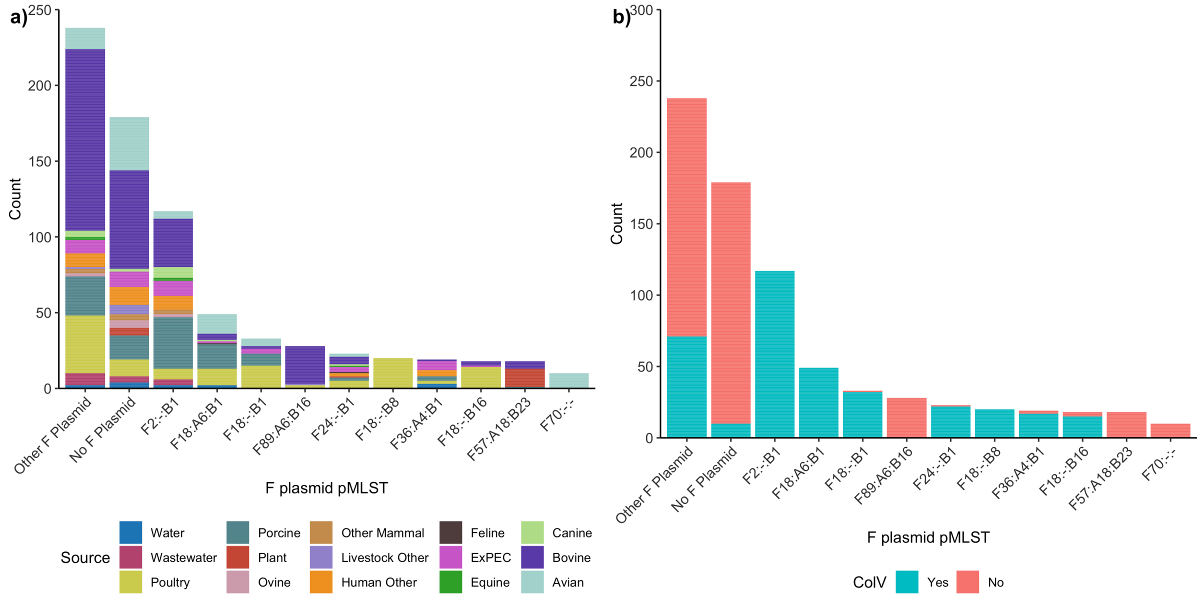

Figure S4. Relationship between F plasmid RST, sources and ColV carriage. Distribution of F plasmid replicon sequence types (RST) stratified by **a)** source and **b)** ColV carriage.

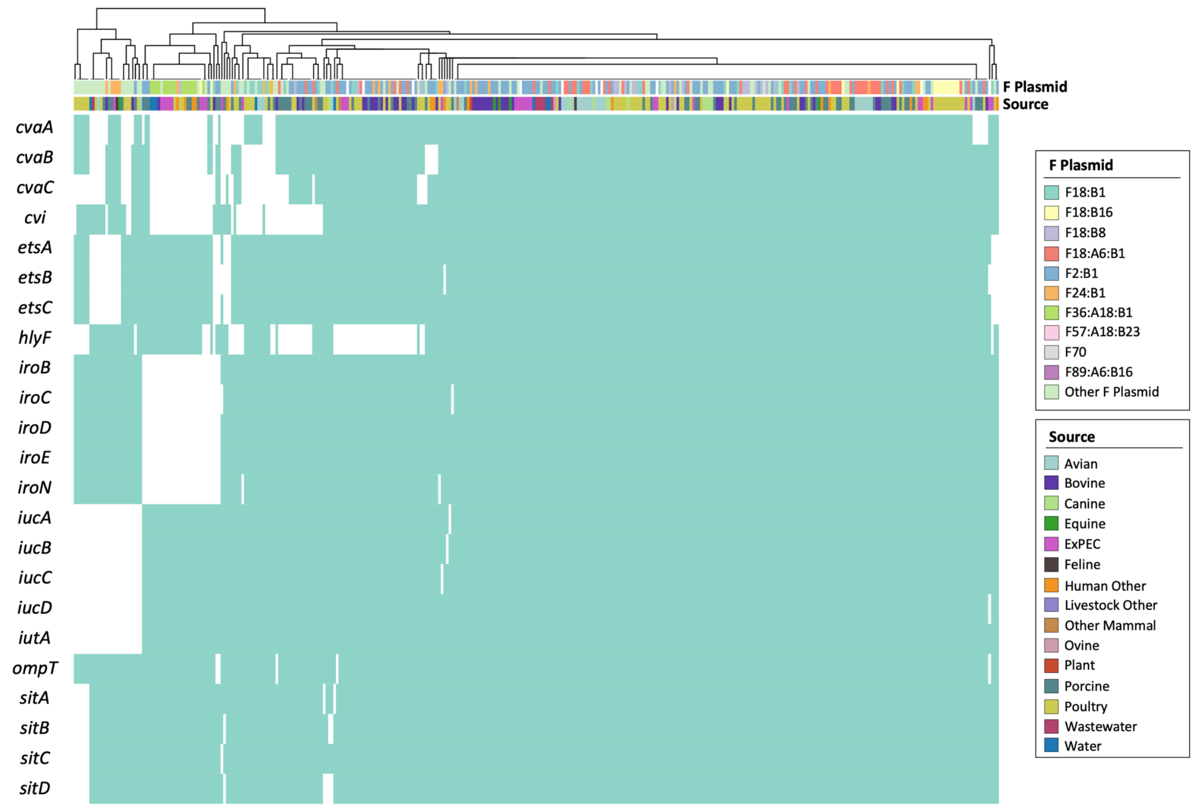

Figure S5. Marker gene carriage in ColV+ ST58 genomes. Presence/absence of ColV typing genes (Liu criteria - see Methods) among 353 ColV+ genome sequences. F plasmid replicon sequence type (RST) and isolate source are shown as metadata bands. Data was hierarchically clustered by gene profile similarity in *pheatmap* 1.0.12.

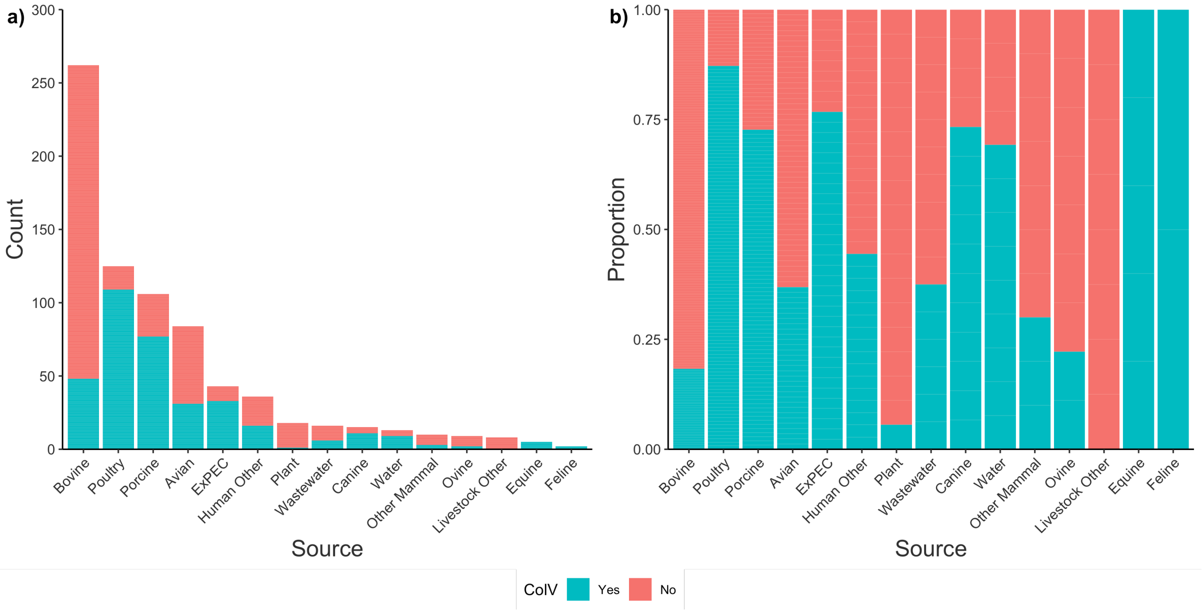

Figure S6. Relationship between source and ColV carriage. Source distribution stratified by **a)** absolute and **b)** proportional ColV carriage.

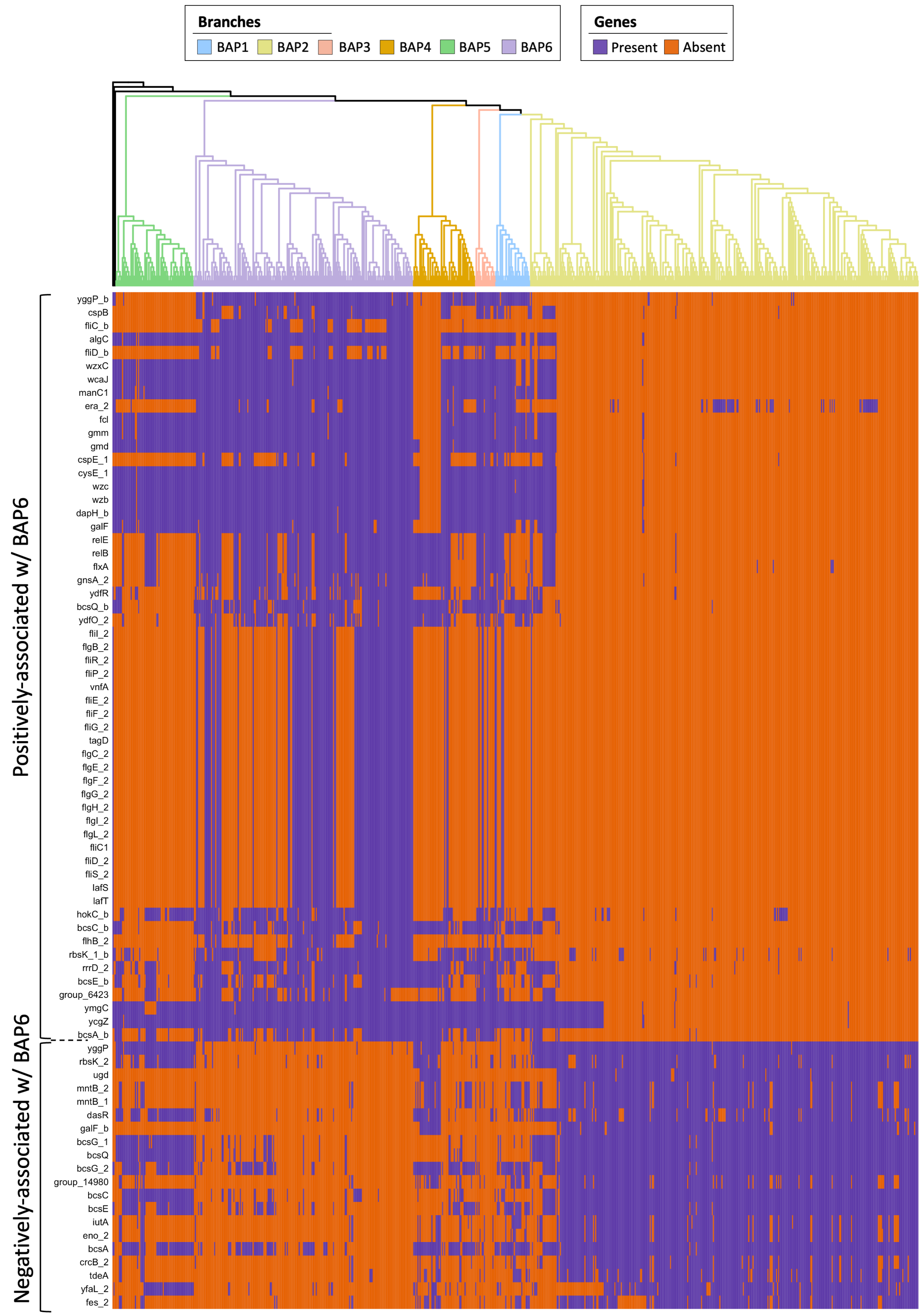

Figure S7. Genes associated with the BAP6 cluster of ST58. Presence/absence (purple/orange) of genes positively and negatively associated with the BAP6 cluster mapped to the phylogeny. Tree branches indicate BAP clusters.

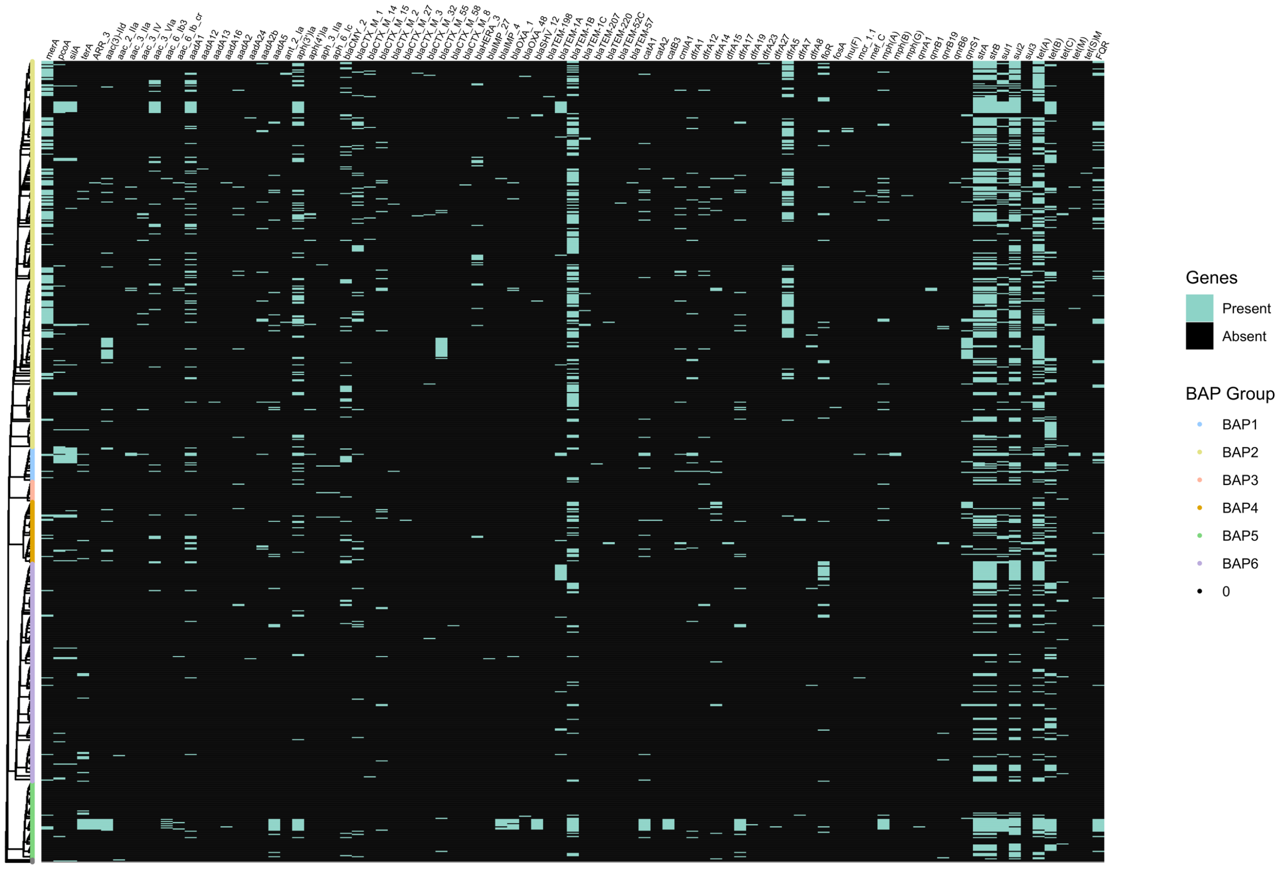

Figure S8. Antimicrobial resistance gene heatmap. Presence/absence (green/black) of antimicrobial and heavy metal resistance genes in 752 ST58 genomes.

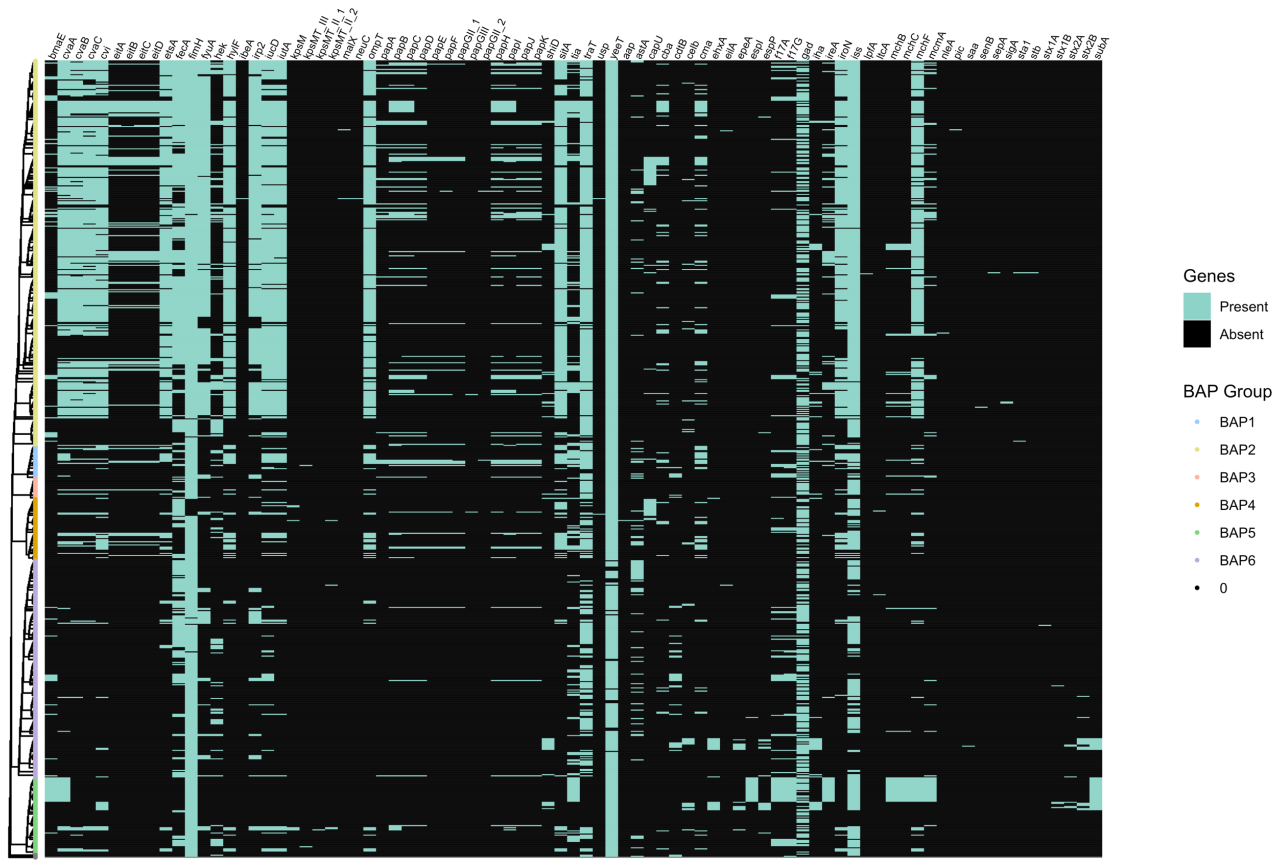

Figure S9. Virulence-associated gene heatmap. Presence/absence (green/black) of virulence-associated genes in 752 ST58 genomes.

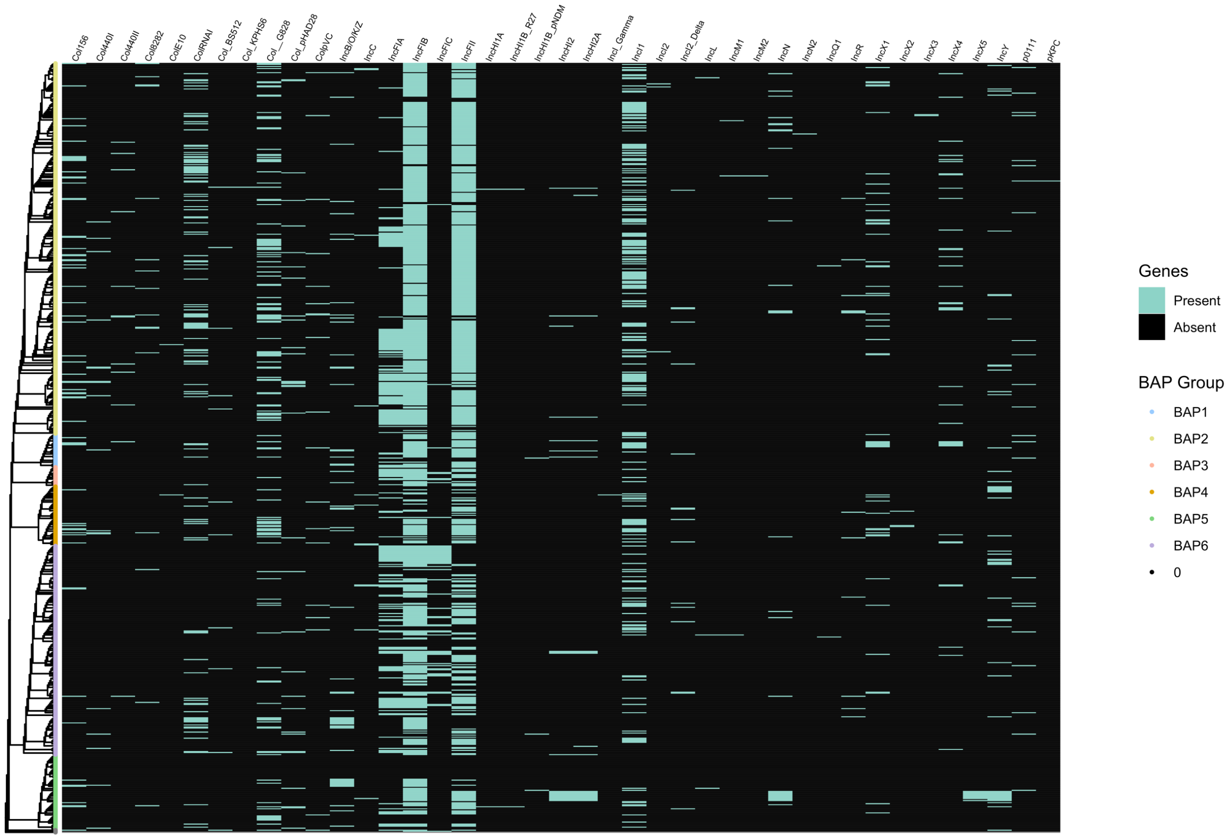

Figure S10. Plasmid replicon gene heatmap. Presence/absence (green/black) of plasmid replicon genes in 752 ST58 genomes.

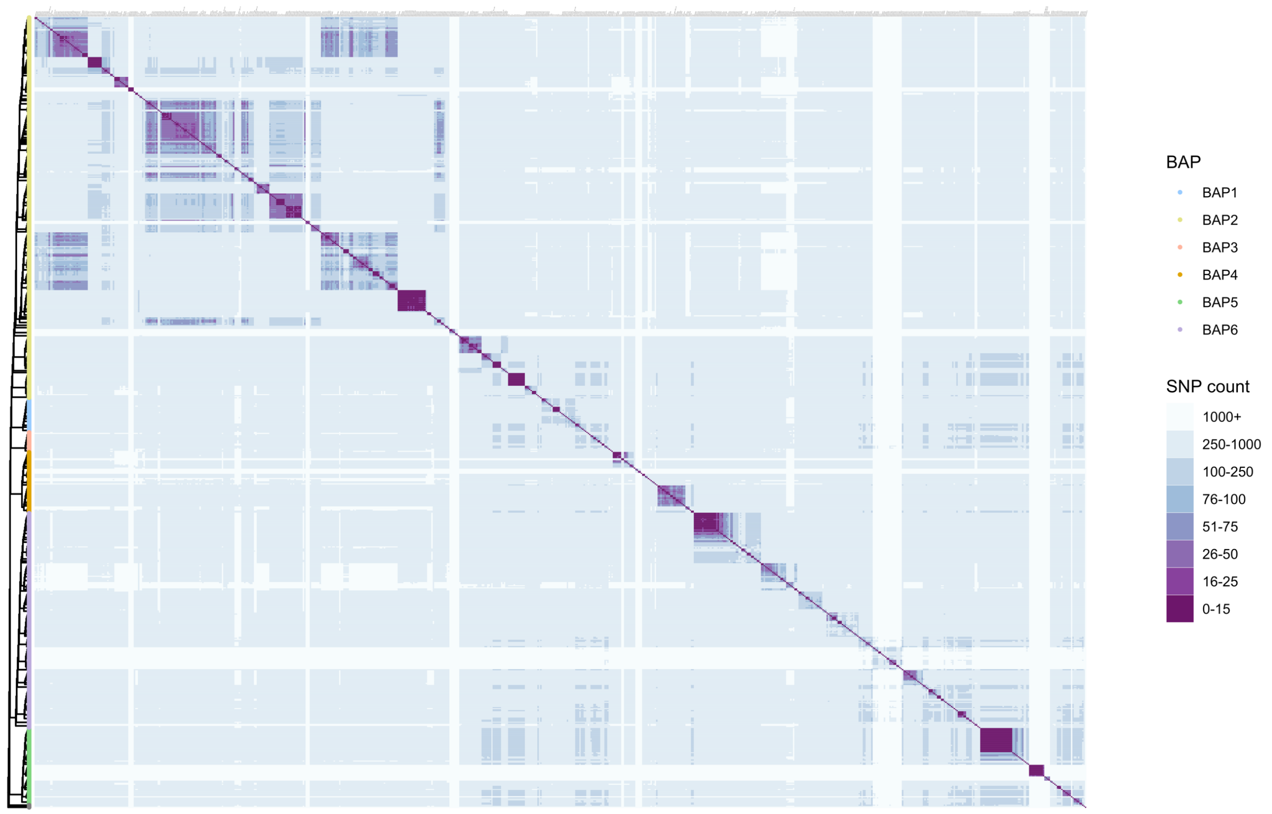

Figure S11. Pairwise SNP distance heatmap. Heatmap of pairwise SNP distances between 752 ST58 genomes mapped to the maximum likelihood phylogeny. Tree branches display BAP colours. Darker squares indicate lower SNP distances.
