# Supplementary material for: A role for ColV plasmids in the evolution of pathogenic Escherichia coli ST58
Source: Nat Commun. 2022 Feb 3;13:683. doi: 10.1038/s41467-022-28342-4 (PMC8813906; doi:10.1038/s41467-022-28342-4)
Supplement: Supplementary file 3 — Description of Additional Supplementary Files [file 41467_2022_28342_MOESM3_ESM.pdf]

Description of Additional Supplementary Files

File Name: Supplementary Data 1
Description: Metadata, accession numbers and assembly statistics for 752 ST58 genomes used in this study

File Name: Supplementary Data 2
Description: Gene screening data for 752 ST58 genomes used in this study

File Name: Supplementary Data 3
Description: Scoary output of genes identified to be significantly over-or under-represented in BAP clusters

File Name: Supplementary Data 4
Description: Metadata and ColV gene screening data for 34,364 *E. coli* genome assemblies from Enterobase
