# Supplementary material for: A role for ColV plasmids in the evolution of pathogenic Escherichia coli ST58
Source: Nat Commun. 2022 Feb 3;13:683. doi: 10.1038/s41467-022-28342-4 (PMC8813906; doi:10.1038/s41467-022-28342-4)
Supplement: Supplementary file 8 — Reporting Summary [file 41467_2022_28342_MOESM8_ESM.pdf]

nature portfolio

Corresponding author(s): Prof. Steven Djordjevic

Last updated by author(s): Dec 10, 2021

Reporting Summary

Nature Portfolio wishes to improve the reproducibility of the work that we publish. This form provides structure for consistency and transparency in reporting. For further information on Nature Portfolio policies, see our Editorial Policies and the Editorial Policy Checklist.

Statistics

For all statistical analyses, confirm that the following items are present in the figure legend, table legend, main text, or Methods section.

n/a	Confirmed	
☐	☒	The exact sample size (n) for each experimental group/condition, given as a discrete number and unit of measurement
☒	☐	A statement on whether measurements were taken from distinct samples or whether the same sample was measured repeatedly
☐	☒	The statistical test(s) used AND whether they are one- or two-sided<br>*Only common tests should be described solely by name; describe more complex techniques in the Methods section.*
☒	☐	A description of all covariates tested
☒	☐	A description of any assumptions or corrections, such as tests of normality and adjustment for multiple comparisons
☒	☐	A full description of the statistical parameters including central tendency (e.g. means) or other basic estimates (e.g. regression coefficient) AND variation (e.g. standard deviation) or associated estimates of uncertainty (e.g. confidence intervals)
☐	☒	For null hypothesis testing, the test statistic (e.g. F, t, r) with confidence intervals, effect sizes, degrees of freedom and P value noted<br>*Give P values as exact values whenever suitable.*
☒	☐	For Bayesian analysis, information on the choice of priors and Markov chain Monte Carlo settings
☒	☐	For hierarchical and complex designs, identification of the appropriate level for tests and full reporting of outcomes
☒	☐	Estimates of effect sizes (e.g. Cohen's d, Pearson's r), indicating how they were calculated

Our web collection on statistics for biologists contains articles on many of the points above.

Software and code

Policy information about availability of computer code

Data collection	ST58 genomes were downloaded from NCBI SRA via parallel-fastq-dump v0.6.6 using SRA and ENA accession numbers listed in Supplementary Data 1. The Enterobase collection was downloaded as assemblies with a custom script available at https://github.com/C-Connor/EnterobaseGenomeAssemblyDownload using Assembly Barcodes listed in Supplementary Data 4.
Data analysis	Software versions: Shovill 1.0.4; prokka 1.14.5; Roary 3.13.0; MAFFT 7.455; IQTree 2.0.3; fastbaps 1.0.6; snp-sites 2.5.1; snp-dists 0.6.3; Scoary 1.6.16; ARIBA 2.13.3; PointFinder 3.1.0; ABRicate 0.9.8.<br>RStudio version: 1.4.1106. R version: 4.0.5. R package versions: data.table_1.14.0, tidyverse_1.3.1, magrittr_2.0.1, RColorBrewer_1.1-2, ggtree_3.1.0, pheatmap_1.0.12, reshape2_1.4.4, ggpubr_0.4.0, ggplot2_3.3.5, tibble_3.1.4, purrr_0.3.4, readr_2.0.1, stringr_1.4.0, forcats_0.5.1, tidyr_1.1.3, dplyr_1.0.7.<br>Please see https://github.com/CJREID/ST58_project for complete data analysis scripts.

For manuscripts utilizing custom algorithms or software that are central to the research but not yet described in published literature, software must be made available to editors and reviewers. We strongly encourage code deposition in a community repository (e.g. GitHub). See the Nature Portfolio guidelines for submitting code & software for further information.

Data

Policy information about availability of data

All manuscripts must include a data availability statement. This statement should provide the following information, where applicable:

- Accession codes, unique identifiers, or web links for publicly available datasets
- A description of any restrictions on data availability
- For clinical datasets or third party data, please ensure that the statement adheres to our policy

All ST58 raw sequence read data generated for the first time in this study have been deposited in NCBI BioProject PRJNA727368 [https://www.ncbi.nlm.nih.gov/bioproject/727368] with individual accession numbers available in Supplementary Data 1. All other ST58 raw sequence read data used in this study are available via either the NCBI Sequence Read Archive [https://www.ncbi.nlm.nih.gov/sra] or the EMBL-EBI European Nucleotide Archive [https://www.ebi.ac.uk/ena/browser/home] via accession numbers listed in Supplementary Data 1. Genome assemblies from the Enterobase collection are available at https://enterobase.warwick.ac.uk/species/index/ecoli via assembly barcodes listed in Supplementary Data 4. Source Data are available with this paper.

Field-specific reporting

Please select the one below that is the best fit for your research. If you are not sure, read the appropriate sections before making your selection.

☐ Life sciences ☐ Behavioural & social sciences ☒ Ecological, evolutionary & environmental sciences

For a reference copy of the document with all sections, see nature.com/documents/nr-reporting-summary-flat.pdf

Ecological, evolutionary & environmental sciences study design

All studies must disclose on these points even when the disclosure is negative.

Study description	The study was a genomic exploration of a convenience sample of 752 E. coli ST58 genomes in order to characterise their source distribution, phylogeny, molecular characteristics and any links between and/or trends within these characteristics.
Research sample	The primary research sample was a convenience collection of 752 ST58 E. coli whole genome paired-end sequencing reads collected by ourselves and collaborators (n=178) and also downloaded from public repositories online (n=574). These data are available in NCBI BioProject PRJNA727368 [https://www.ncbi.nlm.nih.gov/bioproject/727368] with individual accession numbers available in Supplementary Data 1. All other ST58 raw sequence read data are available via either the NCBI Sequence Read Archive [https://www.ncbi.nlm.nih.gov/sra] or the EMBL-EBI European Nucleotide Archive [https://www.ebi.ac.uk/ena/browser/home] via accession numbers listed in Supplementary Data 1.The secondary sample was a convenience collection of 34,364 E. coli genome assemblies from Enterobase [https://enterobase.warwick.ac.uk/species/index/ecoli], selected based on presence of appropriate metadata described in Methods and accessible via Assembly Barcodes listed in Supplementary Data 4.
Sampling strategy	Random. Given the global distribution with regard to geography, host and niche abundance of E. coli ST58 is currently impossible to measure with presently available techniques, we simply tried to include as many genomes as possible.
Data collection	Whole genome sequencing reads were collected from collaborating authors by Cameron J. Reid via file transfer and metadata compiled based on spreadsheets shared by co-authors Michael SM Brwouer, Henrik Hasman, Monika Dolejska, Stefanie Hess and Thomas Berendonk.
Timing and spatial scale	We had no influence over the collection dates or spatial scale of the data collection, we simply compiled as many sequences as we could for both the ST58 and Enterobase E. coli collections respectively. The resulting temporal scale is from 1970-2019 and spatial scale includes six continents; Australasia, North America, South America, Asia, Africa and Europe. The ST58 dataset was downloaded/compiled on 4/12/19 and the Enterobase collection was downloaded from 12/1/20.
Data exclusions	Genomes from the Enterobase genome collection were excluded from analysis if they did not have metadata for source of isolation, country/continent of isolation or date of isolation as this information was required to examine epidemiological questions.
Reproducibility	All genome sequence data are publicly available so analysis on them can be reproduced. Quality of genome sequences is verified by QC processes during upload to public repositories Enterobase and SRA. Primary computational analysis tools used are reproducible given the same sequence data and the same package versions that we have provided. Exceptions include Shovill, Prokka and Roary, which will produce very slightly different results each time they are run, though the overall conclusions that one would draw from their outputs should not be different. All secondary analysis is completely reproducible via R scripts and datasets freely available at https://github.com/CJREID/ST58_project.
Randomization	Entire sample is random due to convenience sampling.
Blinding	Blinding was not relevant as this is not a clinical research study and the sample collection was considered as whole, not divided into treatment groups.

Did the study involve field work? ☐ Yes ☒ No

Reporting for specific materials, systems and methods

We require information from authors about some types of materials, experimental systems and methods used in many studies. Here, indicate whether each material, system or method listed is relevant to your study. If you are not sure if a list item applies to your research, read the appropriate section before selecting a response.

Materials & experimental systems

n/a	Involved in the study
☒	☐ Antibodies
☒	☐ Eukaryotic cell lines
☒	☐ Palaeontology and archaeology
☒	☐ Animals and other organisms
☒	☐ Human research participants
☒	☐ Clinical data
☒	☐ Dual use research of concern

Methods

n/a	Involved in the study
☒	☐ ChIP-seq
☒	☐ Flow cytometry
☒	☐ MRI-based neuroimaging
